# Comparative Study of Microbiological Monitoring Results from Three Types of Sampling Methods after Gastrointestinal Endoscope Reprocessing

**DOI:** 10.1155/2019/7940468

**Published:** 2019-12-03

**Authors:** Su Ma, Lili Feng, Ziyi Jiang, Xian Gao, Xisha Long, Shaonan Zhuang, Wenxia Ding, Taiyao Chen, Zhaoshen Li, Lingjuan Zhang, Huijun Xi, Hongzhi Zhang

**Affiliations:** ^1^Gastrointestinal Endoscopy Center, Changhai Hospital, Shanghai, China; ^2^Department of Anesthesiology, Changhai Hospital, Shanghai, China; ^3^School of Nursing, The Navy Military Medical University, Shanghai, China; ^4^Department of Nursing, Changhai Hospital, Shanghai, China; ^5^Shanghai Municipal Center for Disease Control and Prevention, Shanghai, China

## Abstract

**Objective:**

Compare the effects of three sampling methods on the microbiological monitoring results after reprocessing of gastrointestinal endoscopes, providing scientific basis for improving the monitoring quality of gastrointestinal endoscope cleaning and disinfection.

**Method:**

Gastrointestinal endoscopes after reprocessing were selected randomly at the gastrointestinal endoscopy center of a tertiary hospital in Shanghai from October 2018 to February 2019. The endoscopes selected were all sampled in three different methods under continuous sampling and intermittent sampling respectively. Methods used includes, the biopsy channel group (Group A), the entire channel group (Group B), and the disc brush group (Group C). Then the colony forming units (CFU/piece) were counted in the laboratory.

**Results:**

A total of 12 endoscopes were sampled by using continuous sampling approach, in which the detection rate of bacteria in disc brush group (33.3%) and entire channel group (33.3%) was higher than biopsy channel group (8.3%). Among the 12 endoscopes sampled with intermittent approach, the detection rate of bacteria from high to low was the disc brush group (50%), the entire channel group (41.7%), and the biopsy channel group (8.3%).

**Conclusion:**

Different sampling methods will lead to the difference of microbiological culture results after reprocessing of gastrointestinal endoscope, indicating that the improved sampling method is beneficial to objectively reflect the endoscope cleaning and disinfection effect, and improve the monitoring quality of endoscope disinfection.

## 1. Introduction

In recent years, flexible endoscope reprocessing failure has been listed in the “Top 10 Health Technology Hazards” issued annually by the Emergency Care Research Institute (ECRI) for five consecutive years and even ranked the top one of the list in the year 2016. With the continuous advancement of gastrointestinal endoscopy technology, new endoscopic technique may bring new medical risks and medical technique hazards while improving medical quality and patient safety. According to the report, from 1996 to 2015, among 1389 cases of patients under duodenoscopy procedures in Europe, 32 cases were found of being infected with multidrug resistant *Escherichia coli* due to the failure of endoscopic reprocessing [1]; from October 3, 2014, to January 28, 2015, patients from UCLA Medical Center died of Carbapenem-resistant Enterobacter (CRE) infections obtained from contaminated endoscopy; in 2015, 186 patients were infected with Middle East Respiratory Syndrome due to endoscopic reprocessing failure, of which 19.4% died [2]. As per the data from the American Journal of Infection Control, 2018, endoscopes after reprocessing, including gastroendoscope, intestinal endoscope, duodenal endoscope, ultrasound endoscope, etc., the bacterial positive rate ranges from 60% to 92% [3], and in China from 2007 to 2012, the qualification rate after reprocessing of gastrointestinal endoscopes was only 80.8% [4]. Accordingly, the quality of the gastrointestinal endoscopic reprocessing is not stable, and scientific and objective monitoring is urgently needed. Meanwhile, more and more domestic and international guidelines emphasize the importance of endoscope microbial monitoring.

The microbiological monitoring can evaluate the effect and quality of endoscopic reprocessing, and is beneficial to identify sources of contamination, correct cleaning, and disinfection methods, and thus preventing the spread of nosocomial infections It is mentioned in the guidelines from Europe, Australia, and New Zealand [5]. For microbiological sampling of gastrointestinal endoscope, the APIC (Association for Professionals in Infection Control Epidemiology, 2000) recommends sampling the suction and biopsy channel as well as the air-water channel in a flushing method; the ESGE-ESGENA (European Society of Gastrointestinal Endoscopy and European Society of Gastroenterology and Endoscopy Nurses and Associates, 2008), Canada (2010) sets the rule that the endoscopes should be sampled with entire channel by antegrade flushing method. The GESA-GENCA (Gastroenterological Society of Australia and Gastroenterological Nurses College of Australia, 2010) recommends to sample the entire channel of endoscopes with the sequence of flush-brush-flush method in both antegrade and retrograde manner, and the UMCG (University Medical Center Groningen, 2011) suggests the sampling by retrograde rinsing of endoscopic suction biopsy and air-water channel [6]. In order to improve the detection rate of endoscopic microbial contamination after reprocessing, and to more objectively evaluate the effect of endoscopic reprocessing, the Ministry of Health of China has issued the national standard for endoscope cleaning and disinfection since 2004, established the requirement of sample frequency, channel should be sampled and fluid volume of samples. In 2016, newly issued “Regulation for cleaning and disinfection technique of flexible endoscope WS 507-2016” [7] updates and supplements bunches of quality control methods and details of endoscope cleaning and disinfection. The regulation emphasized the entire channel sample method, using 50 ml instead of 20 ml of eluate containing neutralizer, it also emphasized the total collection method, as well as bacteria culture by filter membrane method to improve the elution effect and detection efficiency. However, there are wide difference on sampling sites, sampling methods, and frequency, as well as evaluation indicators between different international guidelines [8]. It becomes an urgent problem and needs to be tackled on how to conduct a microbiological monitoring examination more scientifically, reasonably, and regularly. This study starts with the sampling method in gastrointestinal endoscopic microbiological monitoring, discusses the influence of different sampling methods on the culture results, and provides basis for further establishing scientific and accurate culture methods. The specific procedures are summarized as follows.

## 2. Materials and Methods

### 2.1. Experimental Material

A total of 12 gastrointestinal endoscopes were randomly selected from October 2018 to February 2019 from the gastrointestinal endoscopy center of a tertiary hospital in Shanghai, included 10 gastroscopes (Olympus, Japan), 2 colonoscopes (Olympus, Japan), and 24 disc brushes (Normandie Endo Technologies, France, general type). The neutralizer is an aldehyde neutralization enrichment medium (Haibo Biotechnology, China).

### 2.2. Experimental Methods

#### 2.2.1. Endoscope Cleaning and Disinfection Method

According to the operation requirements of “Regulation for cleaning and disinfection technique of flexible endoscope WS 507-2016”, every endoscope should be strictly reprocessed in accordance with the procedures of “precleaning, leak testing, washing, rinsing, disinfection, terminal rinsing, and drying”.

#### 2.2.2. Sampling Method

In order to avoid the deviation of the detection results due to the difference in the amount of bacteria contaminated between different endoscopes, two approaches were used in this experiment. One was to perform the biopsy channel sampling, the entire channel sampling, and disc brush sampling continuously on the same gastrointestinal endoscope in the same day for further testing, which was called as continuous sampling; the other was to perform biopsy channel sampling, entire channel sampling, and disc brush sampling on the same endoscope for three days respectively for further testing, which was called as intermittent sampling. The entire operating procedure followed the principle of aseptic technique. A peristaltic pump was used and an injection needle was repeatedly injected for 2-3 times for full amount collection.

**Figure 1:**
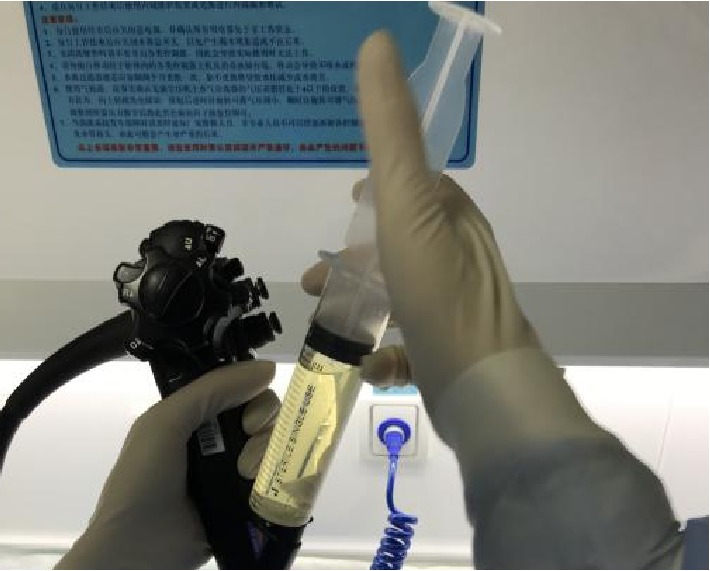
Flush the instrument channel from the control section (Group A).

**Figure 2:**
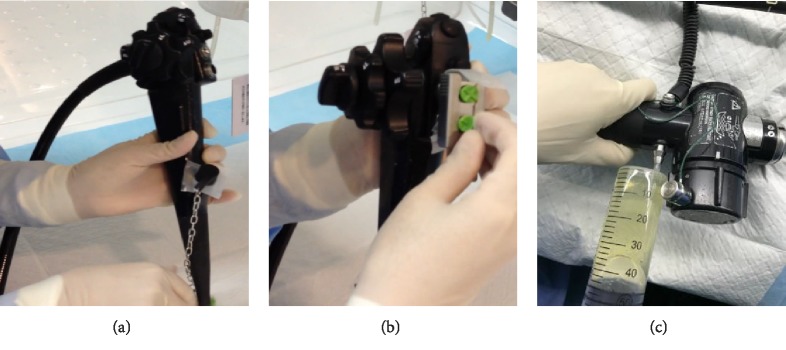
Flush the instrument and suction channel from the suction port (Group B). (a) Close the instrument port, (b) close the air/water and suction port, and (c) flush the suction port.

**Figure 3:**
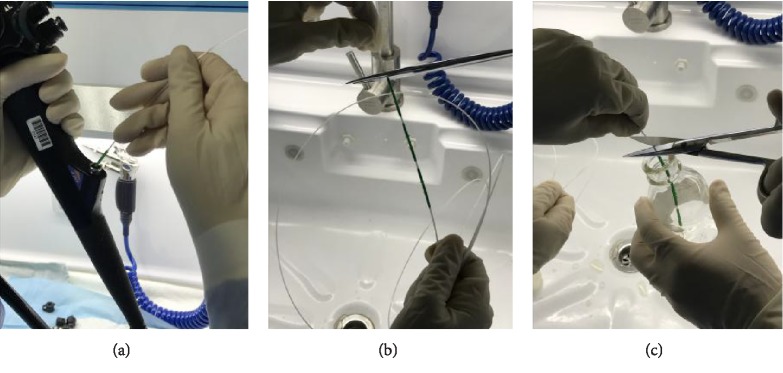
Brush-flush the instrument channel from the instrument port (Group C). (a) Brush the instrument channel, (b) cut off the brush, and (c) cut into the bottle.

1. Biopsy channel sampling group (Group A): the endoscopes after reprocessed were sampled as below: 50 ml of the neutralizer was extracted with a sterile syringe, injected, and flushed the instrument channel through the biopsy port of the control section, and the total volume of elution was collected from the distal end of the endoscope. The eluate was thoroughly mixed and sent to the laboratory of Shanghai Municipal Center for Disease Control and Prevention within 2 hours for culturing and colony counting (CFU/piece) (Figure 1).
2. Entire channel sampling group (Group B): the endoscopes after reprocessed were sampled as below: 50 ml of the neutralizer was extracted with a sterile syringe. A sterile film was placed onto the air/water port, suction, and instrument port of the endoscopic control section. Installed the sterile endoscope specialized washing joint to seal the air/water injection port, suction port; and instrument port of the endoscope control section. The neutralizer was injected and flushed through the endoscope channel from the suction port beside the endoscope light guiding connector, through the suction and biopsy channel and then the total volume of elution was collected from the distal end of the endoscope. The eluate was thoroughly mixed and sent to the laboratory of Shanghai Municipal Center for Disease Control and Prevention within 2 hours for culturing and colony counting (CFU/piece) (Figure 2).
3. Disc brush sampling group (Group C): the endoscopes after reprocessed were sampled as the procedure below: a blunt head of a sterile disposable disc brush was inserted into the instrument port, and the instrument channel was brushed until the brush end completely exited the instrument channel outlet of the distal end. The upper part of the brush was cut off (2 cm) using sterile scissors, then the clipped brush was put into a sterile bottle for testing. 50 ml of neutralizer was extracted with a sterile syringe and injected into the instrument port, then the total volume of elution was collected from the distal end of the endoscope into the same bottle used for the testing of the clipped brush. The eluate was thoroughly mixed with the brush inside the sterile bottle and sent to the laboratory of Shanghai Municipal Center for Disease Control and Prevention within 2 hours for culturing and colony counting (CFU/piece) (Figure 3).

### 2.3. Colony Counts

In reference to the “Hygienic standard for disinfection in hospitals GB 15982-2012”. The test solution was mixed thoroughly with a vortex mixer, inoculated with 1 ml of the mixed eluate in to two plates respectively, 20 ml of the molten nutrient agar medium cooled to 40°C–45°C was poured into each plate, and cultured in an incubator at 35°C for 48 hours, then the number of colonies (CFU/piece) were counted. The remaining eluate (48 ml) was filtered under sterile conditions using a filter membrane (0.45 *μ*m). The inoculate filtered membrane was placed on a solidified nutrient agar plate and cultured in an incubator at 35°C for 48 hours, the number of colonies were counted.

### 2.4. Results Criteria

The cultured results of the samples were recorded. When the filter membrane method is in countable, the total number of colonies (CFU/piece) = *m* (CFU/plate) × 50. When the filter membrane method is countable, the total number of colonies (CFU/piece) = *m* (CFU/plate) + *mf* (CFU/filter membrane). In the formula, “*m*” is the average number of colonies on two parallel plates, and “*mf*” is the number of colonies on the filter membrane. When the colony number of the three methods are 0 CFU/, <1 CFU/ is used to represent the colony number of the three methods. Among them, the result was confirmed as negative if the number of bacterial colonies in the culture results was <1 CFU/piece, and the result was confirmed as positive if ≥1 CFU/piece.

### 2.5. Statistical Methods

The data was analyzed using SPSS 20.0. The statistical methods used are chi-square test for counting data and independent sample Kruskal-Walis test for measurement data.

## 3. Results

In this study, a total of 12 flexible endoscopes were collected using continuous sampling approach, with colony counts ranging from 0 to 21 CFU/piece. The detection rate of bacteria in the disc brush group (33.3%), and the entire channel group (33.3%) was higher than that of the biopsy channel group (8.3%). Among the 12 endoscopes sampled with intermittent approach, with colony counts ranging from 0 to 36 CFU/piece, the detection rate of bacteria from high to low was the disc brush group (50%), the entire channel group (41.7%), and the biopsy channel group (8.3%). It showed that the detection rate of bacteria for either the disc brush group or the entire channel group was higher than the biopsy channel group (Tables 1–4).

## 4. Discussion

### 4.1. Microbiology Culture Is the Gold Standard for Quality Control of Gastrointestinal Endoscope Cleaning and Disinfection

Gastrointestinal endoscopy is an important minimal invasive diagnosis and treatment method for gastrointestinal tract, pancreaticobiliary, and other diseases. It has complex and delicate structure and is difficult to thoroughly reprocess. In recent years, there have been many reports of endoscopy related healthcare associated infections with complex influencing factors. As a reusable medical device that directly contacts with the mucosa of a patient's organs, high-level disinfection, or sterilization must be achieved before use. However, studies have shown that even if the endoscope and accessories are treated strictly in accordance with guideline recommended for cleaning and disinfection methods, endoscopic associated infections are still possible to happen [8], so we believe that no matter the method of cleaning and disinfection, the effect must be verified to ensure the disinfection effect as well as patients' safety. At present, microbiology culture is an important way to evaluate the quality of the endoscope cleaning and disinfection [9].

### 4.2. There Are No Clear Standards and Operating Specifications Regarding Sampling Methods for Endoscope Microbiology Culture

A prospective study of the disinfection effect of a duodenoscope after disinfection and drying in one hospital found that the positive rate was 5–15.5% [10]. A study in Korea found that the positive rate for the treated duodenoscope was 37.2% [11]. Riberiro et al. from Brazil sampled the high-level disinfected endoscopes from 37 healthcare institutions in Minas Gerais, and found that the contamination rate of air-water channel of gastroscope was as high as 70% [12]. It was reported that currently the microbiological sampling methods of endoscopy are focus on antegrade and retrograde way, and the microbial positive rate is higher for the latter one [13–15]. It can be seen that there are wide differences between the results of gastrointestinal endoscopic microbiological cultures from different countries. Different sampling methods will lead to the difference of microbiological culture results, and the false negative results will affect the reliability of the microbiology culture result. Thus, scientific and practical microbiological sampling methods are critical to ensure the quality of flexible endoscope reprocessing.

### 4.3. Positive Detection Rate Can Be Increased by the Disc Brush and the Entire Channel Sampling Method

In the endoscopic microbiology sampling method of our study, the entire channel group sampling was to inject the neutralizer from the instrument port beside the endoscope light guiding connector, and seal the suction port, the air/water injection port and the instrument port of the endoscope control section with the washing joint, and then collect the elution from the distal end of the endoscope. The path taken by the instrument channel sampling group is the same as the brush sampling group. The single biopsy channel was lacking the sampling of the suction channel compared with the entire channel group. However, the disc brush sampling method can brush and wash the inner surface of the endoscope lumen when compared with the simple flushing method of washing off the attachment from the inner surface of lumen. It has been shown that brush sampling method could scrub the endoscope channel and remove separately the new and old contaminants from endoscope channel [16]. It can be seen that the entire channel sampling method is more comprehensive, avoiding the limitations of the other two methods, and the disc brush sampling method could fully scrub the inner wall of the lumen, loosening and washing off the attachments, which might more effectively improve the detection rate of bacteria of endoscopes, but it may also require more staff cooperation between operators. The details, such as the type of brush, type of endoscope, the frequency of brushing practice etc. may need further research in the future [17].

### 4.4. Establishing Standard Microbiological Culture Sampling Method Is the Basis for Improving the Accuracy of Endoscopic Reprocessing Monitor Results

The results of this study shows that the positive rates of the entire channel sampling method and the disc brush sampling method are much higher than the conventional sampling method; whether, by continuous or intermittent approach. The conventional sampling method has been applied in China for nearly 15 years since 2004, and it will continue to be performed by healthcare practitioners in the future. There have been many international guidelines regarding to the endoscopic microbiology sampling, but the lack of well-defined illustrated operational procedures for specific sampling methods is likely to result in denormalization of microbiological sampling and reduced evaluability of results. Therefore, in the future, we can improve the sampling method for endoscopic microbiology culture through continuous research. At the same time, since the positive and negative judgments of the cultured results have no aligned international standard [6], the results of the colony counts in this experiment are grouped according to <1 and ≥1, thus it only represent the detection rate of bacteria instead of whether it is safe to use the endoscopes.

## 5. Conclusion

It was found in this study that both the entire channel sampling and the disc brush sampling method have higher bacterial positive detection rate than the conventional biopsy channel sampling method, which further indicates that the endoscopic sampling method being implemented currently needs further improvement. At the same time, this study also has certain limitations. This study is a single-center study. Meanwhile, the samples we used came from the gastrointestinal endoscopes used in daily clinical practice. The original bioburdens of the endoscope were not under control. Larger sample size and multi-center sites as well as endoscopic simulation models combined with laboratory experiments are needed in the future. Validation and comparison under standard condition could better increase the reliability and scientificity of the study.

## Figures and Tables

**Table 1:** Comparison of bacterial colony counts by different sampling methods (continuous sampling).

Sampling method		Max		Min		*H*		*P*	
Biopsy channel (Group A)		1		0					
Entire channel (Group B)		1		0					
Disc brush (Group C)		21		0					
						2.657		0.265	

**Table 2:** Comparison of bacterial colony counts by different sampling methods (intermittent sampling).

Sampling method		Max		Min		*H*		*P*	
Biopsy channel (Group A)		1		0					
Entire channel (Group B)		36		0					
Disc brush (Group C)		30		0					
						5.626		0.060	

**Table 3:** Comparison of bacterial colony counts group by different sampling methods (continuous sampling).

Sampling method	Colony count group	
	<1 (*n*, %)	≥1 (*n*, %)
Biopsy channel (Group A)	11 (91.7)	1 (8.3)
Entire channel (Group B)	8 (66.7)	4 (33.3)
Disc brush (Group C)	8 (66.7)	4 (33.3)
Total	27 (75)	9 (25)

**Table 4:** Comparison of colony counts group by different sampling methods (intermittent sampling).

Sampling method	Colony counts group	
	<1 (*n*, %)	≥1 (*n*, %)
Biopsy channel (Group A)	11 (91.7)	1 (8.3)
Entire channel (Group B)	7 (58.3)	5 (41.7)
Disc brush (Group C)	6 (50)	6 (50)
Total	24 (66.7)	12 (33.3)

## Data Availability

The data used to support the findings of this study are included within the article.
